# Pulmonary computed tomographic manifestations of COVID-19 in vaccinated and non-vaccinated patients

**DOI:** 10.1038/s41598-023-33942-1

**Published:** 2023-04-27

**Authors:** Esther Askani, Katharina Mueller-Peltzer, Julian Madrid, Marvin Knoke, Dunja Hasic, Christopher L. Schlett, Fabian Bamberg, Prerana Agarwal

**Affiliations:** 1 Department of Diagnostic and Interventional Radiology, Medical Center, University of Freiburg, Hugstetter Str. 55, 79106 Freiburg im Breisgau, Freiburg, Germany; 2 Department of Cardiology, Pneumology, Angiology and Intensive Care, Ortenau Klinikum, Lahr, Germany; 3 Department of Protestant Theology, Faculty of Theology, University of Heidelberg, Heidelberg, Germany

**Keywords:** Clinical trials, Medical research

## Abstract

This study aimed to analyze computed tomographic (CT) imaging features of vaccinated and non-vaccinated COVID-19 patients. The study population of this retrospective single-center cohort study consisted of hospitalized COVID-19 patients who received a chest CT at the study site between July 2021 and February 2022. Qualitative scoring systems (RSNA, CO-RADS, COV-RADS), imaging pattern analysis and semi-quantitative scoring of lung changes were assessed. 105 patients (70,47% male, 62.1 ± 16.79 years, 53.3% fully vaccinated) were included in the data analysis. A significant association between vaccination status and the presence of the crazy-paving pattern was observed in univariate analysis and persisted after step-wise adjustment for possible confounders in multivariate analysis (RR: 2.19, 95% CI: [1.23, 2.62], *P* = 0.024). Scoring systems for probability assessment of the presence of COVID-19 infection showed a significant correlation with the vaccination status in univariate analysis; however, the associations were attenuated after adjustment for virus variant and stage of infection. Semi-quantitative assessment of lung changes due to COVID-19 infection revealed no association with vaccination status. Non-vaccinated patients showed a two-fold higher probability of the crazy-paving pattern compared to vaccinated patients. COVID-19 variants could have a significant impact on the CT-graphic appearance of COVID-19.

## Introduction

At the end of 2019, the SARS-CoV-2 coronavirus caused an infection outbreak in Wuhan, China, leading to a global spread in early 2020, triggering a pandemic that continues today^1,2^. The real-time reverse transcriptase polymerase chain reaction (RT-PCR) testing is considered the gold standard for diagnosing COVID-19 infection^3^. However, in the clinical setting imaging also plays a central role in the early identification of patients potentially suffering from COVID-19^4^. Thus, in the event of a suspected COVID-19 infection and pending RT-PCR test results, imaging features displayed by chest computed tomography (CT) can be decisive in increasing suspicion of COVID-19 infection. This diagnostic work-up can prevent delayed protective measures for healthcare workers and patients. Furthermore, the radiologist may even be the first to express the suspicion of a COVID-19 infection based on imaging features and may help to avoid the COVID-19 virus spread in these cases. Guidance on the classification of CT findings potentially attributable to COVID-19 pneumonia depending on the presence and constellation of specific imaging patterns was published by the Radiological Society of Northern America (RSNA) in March 2020^5^. However, CT imaging features of COVID-19 pneumonia can differ according to the immune status of patients. Thus, it was observed that older patients (> 60 years) with COVID-19 pneumonia had more extensive lung involvement, subpleural lines, pleural thickening, and consolidations compared to younger patients^6,7^. In comparison, CT imaging of younger patients showed more ground glass opacities (GGOs) than older patients^6^.

Since the end of December 2020, vaccines against COVID-19 have been administered in Germany and many other countries^8^. At the time of our study, approximately 74% of the German population had received primary immunization against COVID-19^9^. Based on current knowledge, vaccination protection reduces the likelihood of COVID-19 infection, and a severe course of COVID-19 infection^10^. However, vaccination protection is limited by newly emerging virus variants^11^.

Above mentioned guidance on the classification of CT findings potentially attributable to COVID-19 pneumonia published by the RSNA in March 2020 was written before vaccines were available, using data from non-vaccinated patients at that time^5^.

This study aimed to analyze CT imaging features of vaccinated and non-vaccinated patients hospitalized for COVID-19 and to observe potentially changing imaging features of COVID-19 pneumonia in a setting of steadily increasing vaccination coverage.

## Results

**Figure 1:**
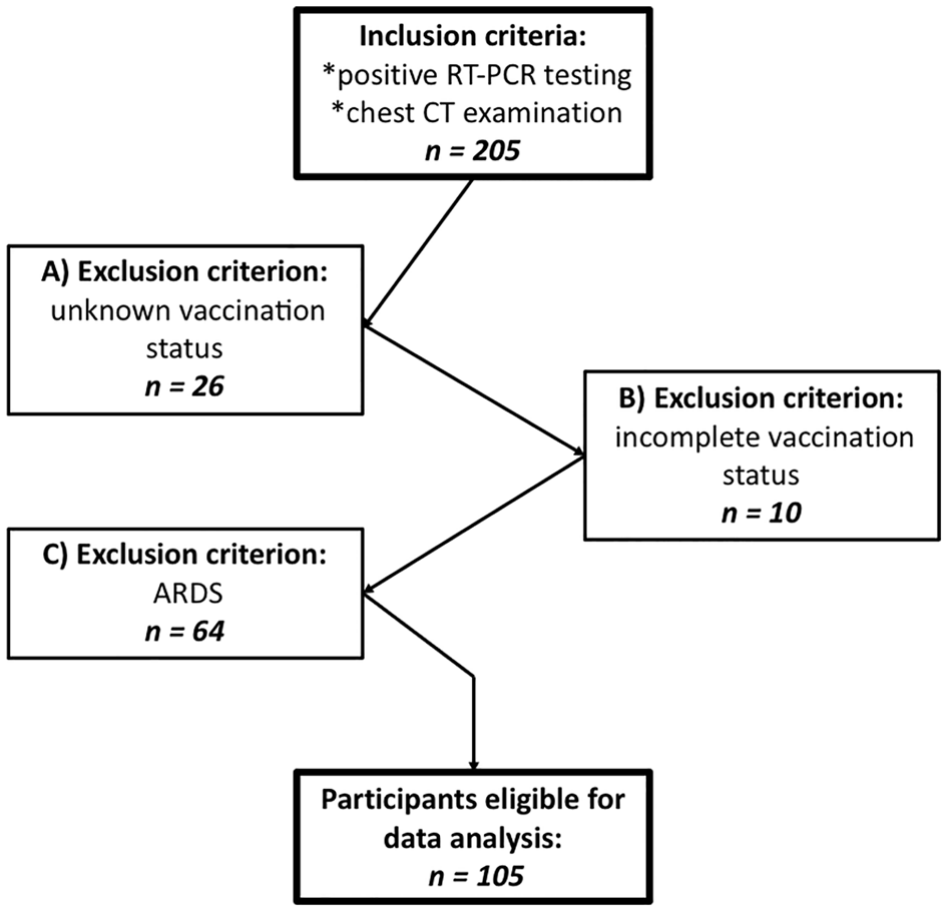
Study Flowchart. Patients’ inclusion and exclusion criteria with representation of included and excluded number of patients.

From July 1, 2021, to February 14, 2022, 205 patients hospitalized due to a COVID-19 infection received a chest CT scan at the study site. Among these, 26 patients were excluded due to unknown vaccination status and 10 because of incomplete vaccination status (out of these partially vaccinated patients, 3 had received a single dose of BNT162b2vaccine-Pfizer-BioNTech, 5 had received one dose of Ad26.COV2.S vaccine-Johnson& Johnson-Janssen, 1 had received a single dose of ChAdOx1 nCoV-19 vaccine-AstraZeneca, and 1 patient had received a single dose of a not specified COVID-19 vaccine). Furthermore, 64 patients were excluded from chest CT analysis because of acute respiratory distress syndrome (ARDS) at the time of the CT scan. Thus, 105 patients (70,47% male, 62.1 ± 16.79 years) were included in the data analysis (Fig. 1).

56 (53.3%) patients were fully vaccinated (out of these fully vaccinated patients, 12 (21.43%) had received two doses of BNT162b2vaccine-Pfizer-BioNTech, 4 (7.14%) had received three doses of BNT162b2vaccine-Pfizer-BioNTech, 3 (5.36%) had received two doses of ChAdOx1 nCoV-19 vaccine-AstraZeneca, 18 (32.14%) had received two doses of a not specified COVID-19 vaccine, and 19 (33.93%) had received three doses of a not specified COVID-19 vaccine).

For 3 out of 105 (2.86%) patients, of whom all had been vaccinated at least twice, a previous COVID-19 infection was reported.

Patients’ demographic data according to vaccination status are presented in Table 1.

**Table 1:** Patients’ demographic and clinical factors according to vaccination status.

	All	Vaccination status		*P*-value
		Non-vaccinated n = 49	Vaccinated n = 56	
General information				
Age (years) (n = 105)	62.1 ± 16.79	54.98 ± 16.11	68.32 ± 14.89	** < 0.001**
Sex (n = 105)				
Male	74 (70.47%)	35 (71.43%)	39 (69.64%)	1.0
Female	31 (29.52%)	14 (28.57%)	17 (30.36%)	
Infection parameters				
Virus variant (n = 60)				
Delta	43 (71.66%)	23 (88.46%)	20 (58.82%)	**0.019**
Omicron	17 (28.33%)	3 (11.54%)	14 (41.18%)	
Symptoms				
Dyspnea (n = 104)	78 (75.00%)	40 (81.63%)	38 (69.09%)	0.176
Cough (n = 100)	58 (58.00%)	25 (52.08%)	33 (63.46%)	0.312
Fever (n = 101)	59 (58.41%)	25 (52.08%)	34 (64.15%)	0.233
Days between symptom onset and hospitalization* (n = 102)	8.67 ± 7.84	8.96 ± 7.11	8.4 ± 8.6	0.357
Pre-existing conditions				
BMI (n = 87)				1.0
< 25 kg/m^2^	34 (39.08%)	16 (40.00%)	18 (38.3%)	
≥ 25 kg/m^2^	53 (60.92%)	24 (60.00%)	29 (61.7%)	
Pre-existing diseases (n = 105)	88 (83.81%)	35 (71.43%)	53 (94.64%)	**0.001**
Immunodeficiency (through disease or medication)	21 (20%)	6 (12.24%)	15 (26.79%)	0.087
Pulmonary disease	26 (24.76%)	6 (12.24%)	20 (35.71%)	**0.007**
Cardiac disease	42 (40%)	11 (22.45%)	31 (55.36%)	** < 0.001**
Hypertension	48 (45.72%)	12 (24.49%)	36 (64.29%)	** < 0.001**
Type 2 diabetes	20 (19.05%)	5 (10.2%)	15 (26.79%)	**0.045**
Oncological disease	24 (22.86%)	5 (10.2%)	19 (33.39%)	**0.005**
Renal disease	30 (28.57%)	7 (14.29%)	23 (41.07%)	**0.003**
Thyroid disease	21 (20%)	10 (20.41%)	11 (19.64%)	1
Treatment of COVID-19-infection				
Pharmacological treatment (n = 105)	70 (66.67%)	35 (71.43%)	35 (62.5%)	0.408
Antiviral drug	4 (3.81%)	3 (6.12%)	1 (1.79%)	0.337
Monoclonal antibodies	45 (42.86%)	25 (51.02%)	20 (35.71%)	0.12
Cortisol	64 (60.95%)	34 (69.39%)	30 /53.57%)	0.112
Oxygen therapy (n = 91)	65 (71.42%)	32 (76.19%)	33 (67.35%)	0.486
Oxygen through Nasal cannula or face mask (n = 87)	60 (68.96%)	31 (73.81%)	29 (64.44%)	0.365
NIV (n = 102)	10 (9.8%)	2 (4.17%)	8 (14.81%)	0.098
High flow oxygen therapy (n = 101)	9 (8.91%)	4 (8.33%)	5 (9.43%)	1
CPAP (n = 97)	4 (4.12%)	2 (4.35%)	2 (3.92%)	1
Intubation (n = 104)	5 (4.8%)	3 (6.12%)	2 (3.64%)	0.665
Tracheotomy (n = 104)	3 (2.88%)	2 (4.08%)	1 (1.82%)	0.6
Intensive care treatment (n = 104)	18 (17.31%)	8 (16.33%)	10 (18.18%)	1.0
Complications				
Pulmonary superinfection (n = 102)	32 (31.38%)	14 (28.57%)	18 (33.96%)	0.67
Pulmonary artery embolism (n = 100)	15 (15%)	5 (10.42%)	10 (19.23%)	0.269
Exitus letalis (n = 104)	8 (7.69%)	3 (6.12%)	5 (9.09%)	0.72
Length of hospital stay (days)** (n = 90)	10.23 ± 10.16	10.48 ± 12.27	10 ± 8.81	0.815

For continuous variables, values are mean ± standard deviation (SD) with p‐values from t-test. For categorical variables, values are counts and percentages with *P*‐values from Fisher's Exact Test.

*BMI* body mass index, *NIV* non-invasive ventilation, *CPAP* continuous positive airway pressure.

Patients developing COVID-19 infection during a hospital stay were excluded from the analysis regarding the days between symptom onset and hospitalization (*); patients with exitus letalis were excluded from the analysis regarding the length of hospital stay (**). Significant *P*-value < 0.05.

Significant values are in bold.

Vaccinated and non-vaccinated patients included in our study did not show a significant difference in pharmacological treatment for COVID-19 infection. The antiviral drug used for treatment was remdesivir, monoclonal antibodies were tocilizumab, REGN-COV2 (casirivimab and imdevimab) and sotrovimab. Furthermore, no difference was found in the timepoint of pharmacological treatment in relation to CT examination between the two groups of vaccinated and non-vaccinated patients; 9 patients received their pharmacological treatment on the days before CT examination (non-vaccinated: 5; vaccinated: 4), 30 patients received their pharmacological treatment on the day of CT examination (non-vaccinated: 14; vaccinated: 16), 17 patients received their pharmacological treatment on the days after CT examination (non-vaccinated: 11; vaccinated: 6) (*P* = 0.456).

34 of 100 patients received their CT at an early stage of infection (non-vaccinated: 9; vaccinated: 25), 22 of 100 patients received their CT at a progressive stage of infection (non-vaccinated: 16; vaccinated: 6), 26 of 100 patients received their CT at a peak stage of infection (non-vaccinated: 16; vaccinated: 10), and 18 of 100 patients received their CT at a late stage of infection (non-vaccinated: 7; vaccinated: 11) (*P* = 0.002).

29 CTs were performed as native CT scans (non-vaccinated: 9; vaccinated: 20), 68 CTs were performed as arterial-phase CT scans (non-vaccinated: 35; vaccinated: 33), and 8 CTs were performed as venous-phase CT scans (non-vaccinated: 5; vaccinated: 3) (*P* = 0.121), depending on the clinical indication.

### Clinical parameters according to vaccination status

In univariate analysis, the virus variant was significantly associated with vaccination status (*P* = 0.019). The delta (B.1.617.2) variant was detected to a higher degree compared to the omicron (B.1.1.529) variant in non-vaccinated patients (delta: 23 (88.46%); omicron: 3 (11.54%)), as well as in vaccinated patients (delta: 20 (58.82%); omicron: 14 (41.18%)).

Age and the presence of pre-existing diseases showed a significant correlation with the vaccination status: vaccinated patients were older than non-vaccinated patients (non-vaccinated: 54.98 ± 16.11 years; vaccinated: 68.32 ± 14.89 years; *P* = 2.566 × 10^–5^), and suffering from pre-existing diseases to a higher degree (non-vaccinated: 35 (71.43%); vaccinated: 53 (94.64%); *P* = 0.001).

The occurrence of symptoms at admission, oxygen supplementation or intensive care treatment, complications during infection, and BMI were not significantly associated with vaccination status. No significant association between vaccination status and exitus letalis was observed; 3 of 49 (6.12%) non-vaccinated patients and 5 of 56 (9.09%) vaccinated patients died in the course of infection.

Patients’ clinical data are presented in Table 1.

### Qualitative scoring, pattern distribution, morphology and vaccination status

**Figure 2:**
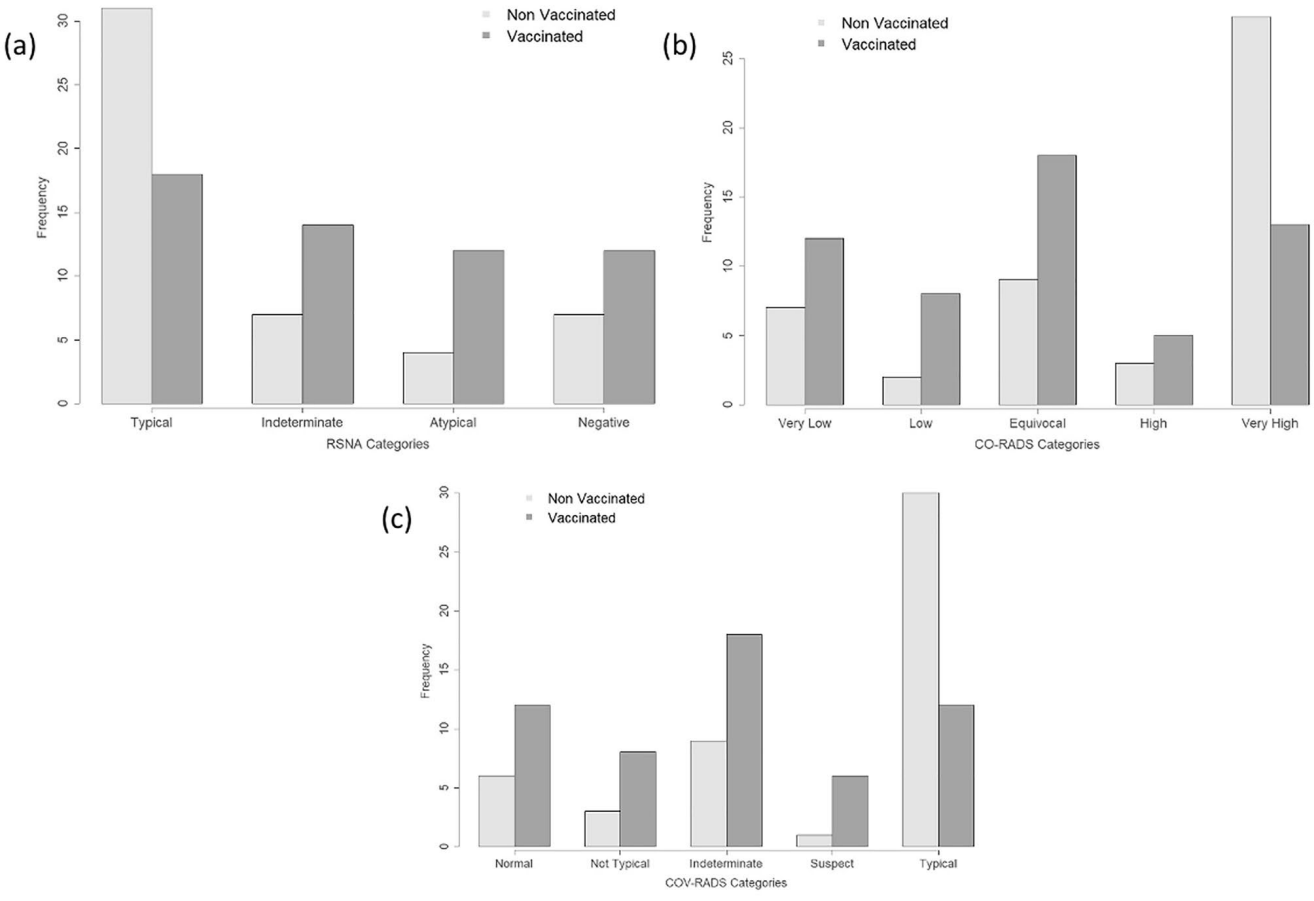
Bar charts. (**a**) Distribution of COVID-19 radiological RSNA scoring system according to vaccination status. *P*-value from Fisher's Exact Test. *P* = 0.014. (**b**) Distribution of COVID-19 radiological CO-RADS scoring system according to vaccination status. *P*-value from Fisher's Exact Test. *P* = 0.008. (**c**) Distribution of COVID-19 radiological COV-RADS scoring system according to vaccination status. *P*-value from Fisher's Exact Test. *P* = 0.001.

In univariate analysis, the applied scoring systems for assessment of the probability of the presence of COVID-19 infection showed a significant correlation with the vaccination status (Table 2, Fig. 2), whereby “typical appearance” and “very high” degree of suspicion were detected more frequently in non-vaccinated than in vaccinated patients (RSNA: *P* = 0.014; CO-RADS: *P* = 0.008; COV-RADS: *P* = 0.001).

**Table 2:** Qualitative scoring-CT-graphic pulmonary manifestation of COVID-19-infection according to vaccination status.

	All (n = 105)	Vaccination Status		*P*-value
		Non-vaccinated (n = 49)	Vaccinated (n = 56)	
RSNA categories				**0.014**
Typical appearance	49 (46.66%)	31 (63.21%)	18 (32.14%)	**0.002**
Indeterminate appearance	21 (20.00%)	7 (14.29%)	14 (25.00%)	0.223
Atypical appearance	16 (15.24%)	4 (8.16%)	12 (21.43%)	0.1
Negative for pneumonia	19 (18.10%)	7 (14.29%)	12 (21.43%)	0.448
CO-RADS categories				**0.008**
Very low	19 (18.10%)	7 (14.29%)	12 (21.43%)	0.448
Low	10 (9.52%)	2 (4.08%)	8 (14.29%)	0.100
Equivocal	27 (25.71%)	9 (18.37%)	18 (32.14%)	0.122
High	8 (7.62%)	3 (6.12%)	5 (8.93%)	0.721
Very high	41 (39.05%)	28 (57.14%)	13 (23.21%)	**0.001**
COV-RADS categories				**0.001**
Normal lung	18 (17.14%)	6 (12.24%)	12 (21.43%)	0.3
Pathological, but not typical for Covid	11 (10.48%)	3 (6.12%)	8 (14.29%)	0.213
Indeterminate	27 (25.71%)	9 (18.37%)	18 (32.14%)	0.122
Suspect of Covid	7 (6.66%)	1 (2.04%)	6 (10.71%)	0.118
Typical	42 (40.00%)	30 (61.22%)	12 (21.43%)	** < 0.001**
Distribution of lung changes				
Axial distribution				**0.023**
No predominant distribution	21 (20.00%)	9 (18.37%)	12 (21.43%)	0.808
Peripheral distribution	51 (48.57%)	31 (63.27%)	20 (35.71%)	**0.006**
Central distribution	9 (8.57%)	3 (6.12%)	6 (10.71%)	0.498
Diffuse distribution	24 (22.85%)	6 (12.24%)	18 (32.14%)	**0.02**
Craniocaudal distribution				0.804
No predominant distribution	19 (18.10%)	7 (14.29%)	12 (21.43%)	0.448
Upper lobe predominant	11 (10.47%)	5 (10.20%)	6 (10.71%)	1
Lower lobe predominant	35 (33.33%)	18 (36.73%)	17 (30.36%)	0.538
Diffuse	40 (38.10%)	19 (38.78%)	21 (37.50%)	1
Other pulmonal findings				
Crazy-paving	23 (21.90%)	18 (36.73%)	5 (8.93%)	**0.001**
Reticulation	26 (24.77%)	11 (22.45%)	15 (26.79%)	0.656
Bronchiectasis	12 (11.43%)	3 (6.12%)	9 (16.07%)	0.134
Bronchial wall thickening	25 (23.81%)	7 (14.29%)	18 (32.14%)	**0.04**
Tree-in-bud	7 (6.66%)	2 (4.08%)	5 (8.93%)	0.445
Bronchoaerogramm	23 (21.90%)	14 (28.57%)	9 (16.07%)	0.157
Vacuolar sign	44 (41.90%)	26 (53.06%)	18 (32.14%)	**0.047**
Reverse halo sign	5 (4.76%)	3 (6.12%)	2 (3.57%)	0.662
COP-pattern	21 (20.00%)	13 (26.53%)	8 (14.29%)	0.145

For categorical variables, values are counts and percentages with *P*‐values from Fisher's Exact Test.

*COP* cryptogenic organizing pneumonia.

Significant *P*-value < 0.05.

Significant values are in bold.

**Figure 3:**
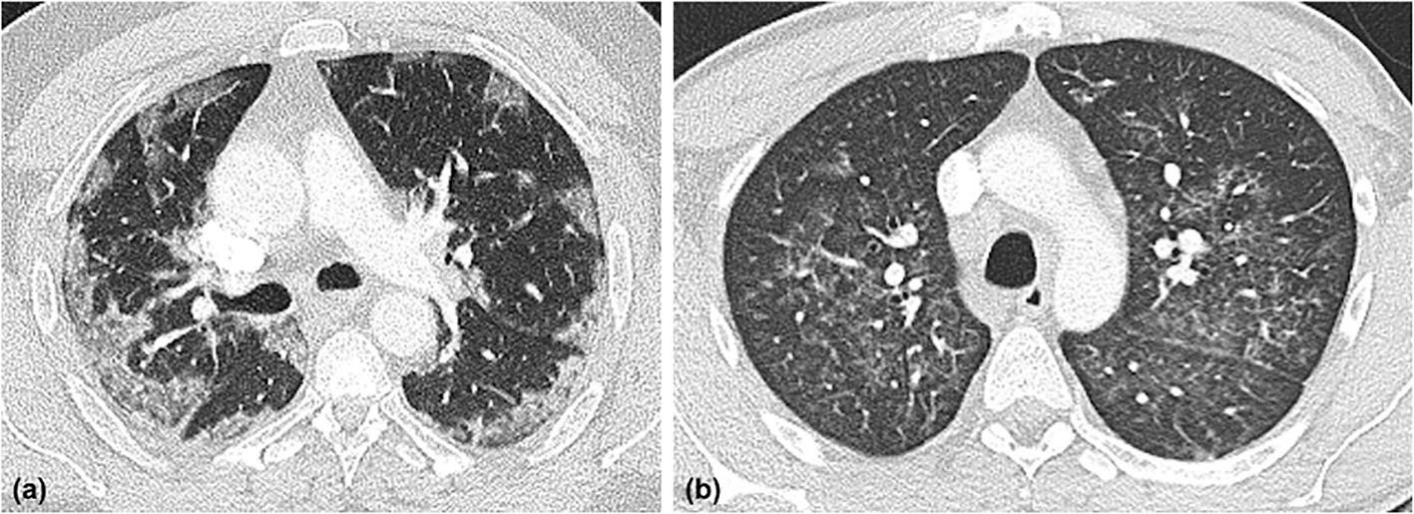
(**a**) 51-year-old non-vaccinated male patient with typical findings of COVID-19 pneumonia, delta-variant: subpleural ground glass opacities (GGO) in a bilateral distribution; (**b**) 37-year-old vaccinated male patient with atypical distribution of COVID-19 pneumonia: peribronchovascular nodular GGO.

Examples of “typical” and “atypical” COVID-19 pneumonia appearance on chest CT are provided in Fig. 3.

However, in multiple logistic regression analysis all associations were attenuated and became non-significant after adjustment for virus variants and stage of infection (Table 3).

**Table 3:** Multiple logistic regression analysis of association of CT-graphic pulmonary manifestations and vaccination status.

Outcome	Vaccination status		*P*-value
	Non-Vaccinated [ref: Vaccinated]		
	RR	95%CI	
Model 1: Association of CT-graphic pulmonary manifestations and vaccination status, adjusted for age and pre-existing diseases			
RSNA categories			
Typical appearance vs all other categories	1.32	[1.07, 1.46]	**0.017**
CO-RADS categories			
Very high vs all other categories	1.43	[1.13, 1.61]	**0.008**
COV-RADS categories			
Typical vs all other categories	1.44	[1.23, 1.56]	**0.001**
Distribution and pattern predominance			
Axial distribution			
Peripheral distribution vs all other categories	1.32	[1.07, 1.53]	**0.017**
Crazy-paving	2.19	[1.56, 2.55]	**0.001**
Bronchial wall thickening	0.84	[0.29, 2.05]	0.728
Vacuolar sign	1.29	[0.90, 1.59]	0.139
Model 2: Model 1 + adjusted for virus variant [delta (B.1.617.2), omicron (B.1.1.529)]			
RSNA categories			
Typical appearance vs all other categories	1.27	[0.84, 1.49]	0.189
CO-RADS categories			
Very high vs all other categories	1.3	[0.75, 1.61]	0.268
COV-RADS categories			
Typical vs all other categories	1.48	[1.20, 1.60]	**0.016**
Distribution and pattern predominance			
Axial distribution			
Peripheral distribution vs all other categories	1.3	[0.90, 1.50]	0.127
Crazy-paving	2.32	[1.50, 2.65]	**0.006**
Bronchial wall thickening	0.89	[0.20; 2.85]	0.869
Vacuolar sign	1.23	[0.62, 1.66]	0.458
Model 3: Model 2 + adjusted for stage of infection [CT scan after symptom onset: peak stage (0-5d), progressive stage (5-8d), peak stage (9-13d), late stage (≥ 14d)]			
RSNA categories			
Typical appearance vs all other categories	1.16	[0.57, 1.47]	0.559
CO-RADS categories			
Very high vs all other categories	1.23	[0.61, 1.60]	0.443
COV-RADS categories			
Typical vs all other categories	1.43	[0.98, 1.59]	0.063
Distribution and pattern predominance			
Axial distribution			
Peripheral distribution vs all other categories	1.24	[0.76, 1.48]	0.281
Crazy-paving	2.19	[1.23, 2.62]	**0.024**
Bronchial wall thickening	1.08	[0.25; 3.31]	0.903
Vacuolar sign	0.96	[0.31, 2.97]	0.922

Results of a logistic regression model with outcome pulmonary changes and exposure vaccination status [ref: Vaccinated].

*RR* relative risk, *CI* confidence interval.

Significant *P*-value < 0.05.

Significant values are in bold.

Evaluation of distribution and pattern predominance showed significant differences of axial distribution in univariate analysis (*P* = 0.023) with a significantly higher degree of peripheral distribution in non-vaccinated individuals (non-vaccinated: 31 (63.27%); vaccinated: 20 (35.71%); *P* = 0.006) and a significantly higher degree of diffuse distribution in vaccinated than in non-vaccinated patients (non-vaccinated: 6 (12.24%); vaccinated: 18 (32.14%); *P* = 0.02) (Table 2). However, the results were no longer significant in multiple logistic regression after adjustment for virus variants (Table 3).

The assessment of other pulmonary findings revealed a significant association between vaccination status and the presence of the crazy-paving pattern (non-vaccinated: 18 (36.73%); vaccinated: 5 (8.93%); *P* = 0.001) as well as the presence of the vacuolar sign (non-vaccinated: 26 (53.06%); vaccinated: 18 (32.14%); *P* = 0.047) and the presence of bronchial wall thickening (non-vaccinated: 7 (14.29%); vaccinated: 18 (32.14%); *P* = 0.04) in univariate analysis (Table 2).

**Figure 4:**
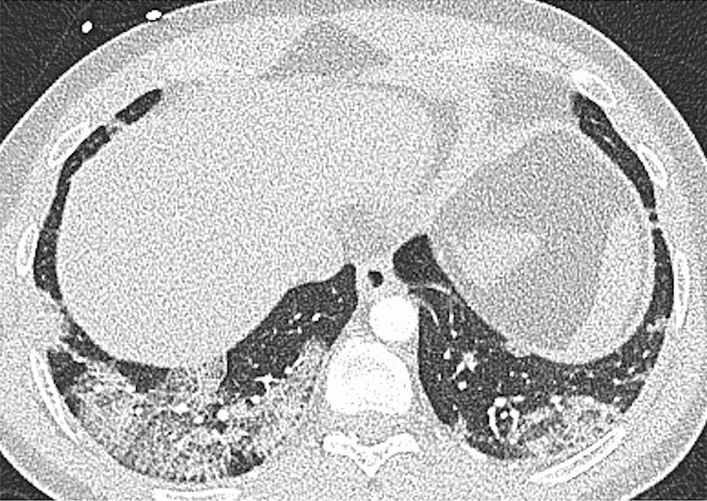
40-year-old non-vaccinated male patient with a typical crazy-paving pattern, well appreciated in the right lower lobe.

While the association of vaccination status with the vacuolar sign and bronchial wall thickening, respectively, became non-significant after adjustment for age and pre-existing diseases, the association between vaccination status and the presence of the crazy-paving pattern persisted in multiple logistic regression analysis after adjustment for age, pre-existing diseases, virus variant and stage of infection with two-fold higher risk for non-vaccinated patients for the presence of the crazy-paving pattern (Model 3: RR: 2.19, 95% CI: [1.23, 2.62], *P* = 0.024) (Table 3). An example of the crazy-paving pattern is provided in Fig. 4.

### Semi-quantitative scoring and vaccination status

**Figure 5:**
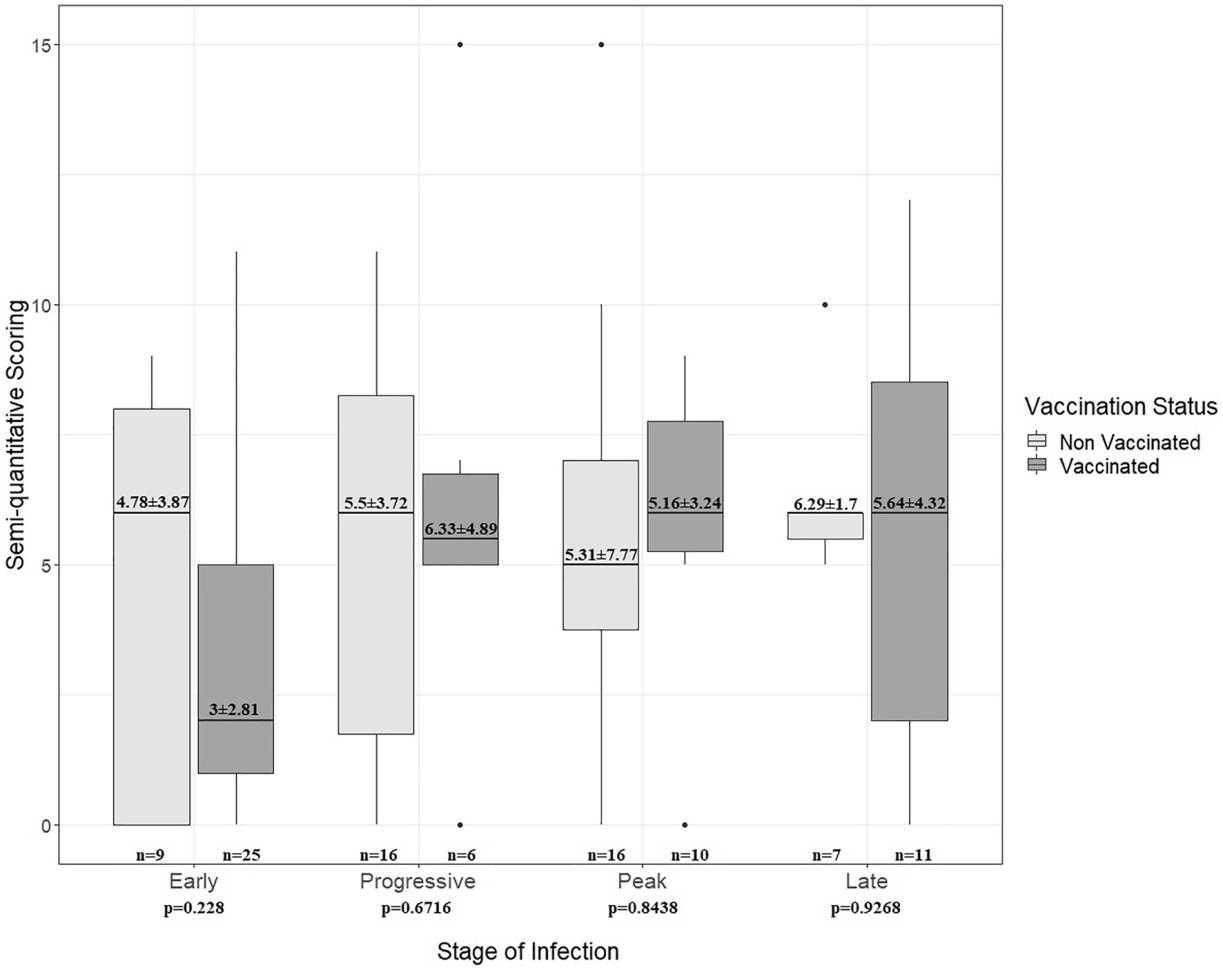
Semi-quantitative scoring: CT-graphic pulmonary manifestation of COVID-19 infection per vaccination status and stage of infection. For continuous variables, values are mean ± standard deviation (SD) with p‐values from Mann–Whitney-U-test (Wilcoxon-rang-sum-test) or from t-test, where appropriate. Early stage (0–5 days after symptom onset), progressive stage (5–8 days after symptom onset), peak stage (9–13 days after symptom onset), late stage (≥ 14 days after symptom onset).

The semi-quantitative assessment of lung changes due to COVID-19 infection revealed no association with the vaccination status regarding “total distribution.” Although initial analysis showed significant results for the “distribution of right upper lobe” changes between non-vaccinated and vaccinated individuals (*P* = 0.045; Table 4), no significant differences were found in the more detailed analysis of semi-quantitative lung involvement according to vaccination status and stage of infection (Fig. 5).

**Table 4:** Semi-quantitative scoring: CT-graphic pulmonary manifestation of COVID-19-infection according to vaccination status.

	All *(n* = *105)*	Vaccination Status		*P*-value
		Non-vaccinated *(n* = *49)*	Vaccinated *(n* = *56)*	
Total distribution				
Semi-quantitative scoring (mean ± SD)	4.9 ± 3.57	5.33 ± 3.5	4.54 ± 3.63	0.203
Distribution right upper lobe				**0.045**
Absent	39 (37.15%)	15 (30.61%)	24 (42.86%)	0.228
< 1/3	48 (45.71%)	22 (44.90%)	26 (46.43%)	1
1/3–2/3	12 (11.42%)	10 (20.41%)	2 (3.57%)	**0.012**
> 2/3	6 (5.71%)	2 (4.08%)	4 (7.14%)	0.683
Distribution right middle lobe				0.539
Absent	41 (39.04%)	18 (36.73%)	23 (41.07%)	0.692
< 1/3	46 (43.81%)	24 (48.98%)	22 (39.29%)	0.332
1/3–2/3	15 (14.28%)	5 (10.20%)	10 (17.86%)	0.403
> 2/3	3 (2.85%)	2 (4.08%)	1 (1.79%)	0.598
Distribution right lower lobe				0.507
Absent	27 (25.72%)	11 (22.45%)	16 (28.57%)	0.510
< 1/3	42 (40.00%)	22 (44.90%)	20 (35.71%)	0.425
1/3–2/3	30 (28.57%)	12 (24.49%)	18 (32.14%)	0.516
> 2/3	6 (5.71%)	4 (8.16%)	2 (3.57%9	0.414
Distribution left upper lobe				0.090
Absent	36 (34.29%)	12 (24.49%)	24 (42.86%)	0.064
< 1/3	40 (38.09%)	23 (46.94%)	17 (30.36%)	0.107
1/3–2/3	22 (20.95%)	9 (18.37%)	13 (23.21%)	0.634
> 2/3	7 (6.66%)	5 (10.20%)	2 (3.57%)	0.247
Distribution left lower lobe				0.231
Absent	27 (25.71%)	9 (18.37%)	18 (32.14%)	0.122
< 1/3	47 (44.76%)	23 (46.94%)	24 (42.86%)	0.698
1/3–2/3	25 (23.81%)	15 (30.61%)	10 (17.86%)	0.169
> 2/3	6 (5.71%)	2 (4.08%)	4 (7.14%)	0.683
GGO scoring				
Semi-quantitative scoring (mean ± SD)	3.54 ± 3.09	3.88 ± 3.19	3.25 ± 3.01	0.382
Consolidation scoring				
Semi-quantitative scoring (mean ± SD)	1.84 ± 2.25	2.24 ± 2.54	1.48 ± 1.92	0.148

For continuous variables, values are mean ± standard deviation (SD) with p‐values from Mann–Whitney-U-test (Wilcoxon-rang-sum-test).

*GGO* ground glass opacity.

Significant *P*-value < 0.05.

Significant values are in bold.

### Inter- and intrareader variability

Interreader and intrareader agreement showed high reliability for all assessed parameters (Krippendorff’s alpha coefficient for interreader variability: RSNA score with α = 0.891, CO-RADS with α = 0.800, COV-RADS with α = 0.842, "total distribution" with α = 0.831; Krippendorff’s alpha coefficient for intrareader variability: RSNA score with α = 0.893, CO-RADS with α = 0.804, COV-RADS with α = 0.849, "total distribution" with α = 0.94).

## Discussion

This retrospective single-center cohort study analyzed the CT-graphic features of vaccinated and non-vaccinated patients hospitalized for COVID-19 from July 1, 2021, to February 14, 2022. 53.3% of 105 patients with COVID-19 were fully vaccinated (minimum of two vaccines).

We found that non-vaccinated patients showed a two-fold higher risk of the crazy-paving pattern—a pattern consisting of scattered or diffuse ground-glass attenuation with superimposed interlobular septal thickening and intralobular lines. The pattern was often described in the initial wave of COVID-19^12^. Non-specific, crazy-paving pattern has been shown to correlate histopathologically with intra-alveolar fibrinous exudates, intra-alveolar macrophages, and interstitial oedema, and has been implicated as a reflection of cytokine storm causing acute lung injury in patients with COVID-19 pneumonia^13,14^. Thus, our results may indicate that vaccination could protect from acute lung injury to some extent. However, further research is needed to elucidate the role of vaccination in individual response at a cellular level which could contribute to a difference in radiological manifestation.

Interestingly, further significant correlations found in univariate analysis, such as “typical appearance” and “very high degree of suspicion,” assessed by the use of different scoring systems for radiological classification of the probability of COVID-19 infection^5,15,16^, were attenuated after adjustment for virus variant and stage of infection in multivariable analysis. Equally interesting is that we did not detect any difference between non-vaccinated and vaccinated patients in relation to the total distribution of lung changes due to COVID-19 infection assessed by a semi-quantitative scoring system.

Since the start of vaccinations against COVID-19, available in Germany since the end of December 2020^8^, only sporadic so-called breakthrough infections were reported initially^17^. However, since the emergence of the delta variant, an increase in breakthrough infections has been recorded^18^. Radiological classification systems for evaluating pulmonary infiltrates in relation to the likelihood of the presence of COVID-19 infection were developed using data before the availability of vaccines^5,15,16^, and thus, they do not account for possible CT-graphic differences of breakthrough infections. However, knowledge about possible differences in CT imaging features of breakthrough infections and primary COVID-19 infections is critical for radiologists so that potential diagnostic delays and delays in taking appropriate protective measures for healthcare workers and patients may be avoided in the acute setting^19^.

Recently, the number of radiological studies on breakthrough infections and the influence of vaccination on pneumonia severity, extent of lung involvement, COVID-19 appearance on chest CT and CT patterns has increased. In one of the first studies focusing on the subject, Lee et al. examined the clinical characteristics, imaging features, and clinical outcomes of patients hospitalized for COVID-19, who had been fully, partially, or not vaccinated in a multicenter cohort^20^. It was found that the proportion of pneumonia-negative CT scans during hospital stays was significantly greater for fully vaccinated patients than non-vaccinated patients. This was also observed by Carbonaro et al., who found that symptomatic COVID-19 patients with a complete vaccination cycle had much higher odds of showing a negative CT chest examination compared to non-vaccinated patients^21^. Similarly, various studies reported higher disease severity assessed by CT severity scores in non-vaccinated patients and milder COVID-19 pneumonia on CT scans in vaccinated patients^22–25^. However, none of these studies considered virus variants, stage of infection, pharmacological treatment or reinfections as possible confounders. Only in one study, no statistically significant differences in CT severity score based on vaccination status were described^26^. While these results are in line with our findings, here as well, results were not adjusted for virus variants. Thus, our results emphasize that virus variants may have a significant impact on the severity of lung involvement in COVID-19 infection. Matching these overall findings, Crombé et al. found that both the omicron variant and vaccination were associated with lesser extent of disease^27^. While significant differences in CT imaging patterns were not observed by Lee et al.^20^ and Carbonaro et al.^21^, Polyakov et al.^28^ analyzed that the overall rate of true-positive findings on CT images rated as typical appearance was lower in vaccinated versus non-vaccinated patients. Perhaps mirroring the findings of Crombé et al.^27^, who found GGO and intralobular reticulations to be less frequent in vaccinated patients, we found a significant difference in the occurrence of the crazy-paving pattern being more frequent in non-vaccinated patients. Geographic or local differences in infection dynamics may explain differences in results. Interestingly, our study cohort covers a very similar time span from July 2021 to February 2022 as the study cohorts investigated by Crombé et al. (from July 2021 to March 2022) and Polyakov et al. (from January 2021 to January 2022); in contrast, Lee et al. (from June to August 2021) and Carbonaro et al. (from May to November 2021) observed their study cohorts for shorter periods. The advantage of covering an extended period is the gain in information as multiple virus variants are observed. The disadvantage is that virus variants may act as possible confounders of primary findings. However, our results were adjusted for virus variants, canceling out these probable confounding effects. Our findings regarding the qualitative scoring systems are engaging in this respect. Significant correlations between qualitative scoring systems and vaccination status could be observed in univariate analysis. As also described by Crombé et al.^27^ and Polyakov et al.^28^, the non-vaccinated patients were likelier to show a typical radiological appearance in the RSNA and COV-RADS categories and a very high degree of suspicion in the CO-RADS categories. Furthermore, although not statistically significant, GGO semi-quantitative scoring revealed a higher prevalence of GGO in non-vaccinated patients. It can be hypothesized that this finding may be a further confirmation of the higher frequency of “typical appearance” in this subgroup, since GGO has been described as the main finding of COVID-19 on CT^5,15,16^. However, previous correlations were attenuated in multivariate analysis mainly after adjustment for virus variants, indicating that virus variants may also significantly influence the radiological appearance of COVID-19 on chest CT. The other variables supporting this hypothesis, namely the vacuolar sign, bronchial wall thickening, and axial distribution, lost their significant associations with vaccination status after adjusting for the virus variant in multivariate analysis. By adjusting for virus variants, we avoided another possible confounder: the significant association between the COVID-19 variant and vaccination status. Two COVID-19 variants were observed in our study—Delta (B.1.617.2) and Omicron (B.1.1.529). In vaccinated patients, a higher rate of the Omicron (B.1.1.529) variant was observed because Omicron (B.1.1.529) occurred at a later time point than Delta (B.1.617.2)^29^, and more individuals had already been vaccinated at this later point in time.

When interpreting chest CTs of COVID-19 patients, radiologists must exercise caution since CT imaging features vary depending on the stage of infection^30^. To overcome this problem, we adjusted the results in multivariate analysis for the stage of infection, and significant initial correlations between vaccination status and COV-RADS were no longer significant.

In our study cohort, vaccinated patients were older and had pre-existing diseases to a greater extent; this is presumably because these groups were triaged among others in the prioritization strategy of initial COVID-19 vaccination distribution^31^.

Interestingly, we did not detect any difference in the semi-quantitative total distribution of lung involvement between non-vaccinated and vaccinated patients after adjustment for the stage of infection. In particular, we found no difference between non-vaccinated and vaccinated patients in the proportion of pneumonia-negative CT scans as described by Lee et al.^20^. However, caution is advised in interpreting these results, which cannot be generalized as the study was conducted on hospitalized COVID-19 patients. To prove the results, a study would have to be conducted on outpatient care on patients who present with the initial symptoms of COVID-19 and are tested positive. One possible explanation for the different outcomes between Lee et al. and our results is that justifying indications vary across countries and even between individual hospitals. In our facility, patients received a chest CT scan only in case of clinical relevance or therapeutic consequences, such as suspicion of pulmonary embolism.

A major advantage of our study is the choice of our study cohort. Thus, only patients who received at least basic immunization, at least two vaccines, were categorized as vaccinated. Partially vaccinated patients were excluded from analysis due to the difficulty in assigning this group properly: on one side, protection against COVID-19 infection and symptomatic disease conferred by two vaccine doses has been reported to be higher than by partial vaccination^32^, on the other side, the advantages of partial vaccination have also been reported, such as reduction of postoperative COVID-19 infection and postoperative mortality^33^. Furthermore, in our study, only a small sample size of 10 patients was recorded as “partially vaccinated,” and excluded from the analysis.”

No difference was observed between patients regarding their treatment during hospitalization. Therefore, we do not assume bias of different therapeutic strategies on CT-graphic appearance. Also, since we only recorded sporadic reinfections in fully vaccinated patients in our study cohort, we do not assume a bias due to previous history of COVID-19 infection.

A few limitations of our study have to be considered. Our sample size is moderate. However, we observed two groups of non-vaccinated and vaccinated patients of similar size (46.7% non-vaccinated, 53.3% vaccinated). Furthermore, we had to exclude individual, incomplete data sets regarding the vaccination status from the analysis. Regarding the generalizability of our results, cautiousness has to be employed since we included only hospitalized COVID-19 patients. So, mild or asymptomatic infections were not considered in our data analysis. Qualitative and semi-quantitative scoring systems may be prone to subjective errors. However, inter- and intrareader reliability were examined and showed high reliability. Due to the moderate sample size and missing data, we were not able to control for variables such as time since vaccination and vaccination type.

In conclusion, our data provide evidence of a difference in CT imaging patterns between non-vaccinated and vaccinated COVID-19 patients. In particular, the crazy-paving pattern was significantly associated with vaccination status regardless of COVID-19 variants and stage of infection and was observed to a higher degree in non-vaccinated patients. In addition, our data may indicate that COVID-19 variants could significantly impact the CT-graphic appearance of COVID-19. Further studies are needed to assess the influence of COVID-19 variants on CT imaging patterns. Based on our results, the alertness of radiologists may be directed to potentially changing computed tomographic manifestations of COVID-19, given the steady increase in vaccination rates. In a dynamic infection process, as we are experiencing with COVID-19, it remains essential to update our scientific state of research constantly. In addition, the mechanisms leading to different parenchymal changes in vaccinated individuals with COVID-19 infection remain to be investigated.

## Materials and methods

### Study design and study population

The study population of this retrospective single-center cohort study consisted of patients with COVID-19 infection who had been hospitalized in a maximum care hospital. Vaccination status served as exposure of interest, and CT-graphic pulmonary manifestations of COVID-19 infection served as outcome variables. Regular recording of in-patient vaccination history at the study site began in July 2021. Patients receiving a chest CT performed within the study time window between July 1, 2021, and February 14, 2022, were consecutively included in the study.

Inclusion criteria were a confirmed COVID-19 infection with at least one positive RT-PCR testing of nasal or throat swab and at least one chest CT examination during hospitalization. Exclusion criteria were missing data on vaccination status, partial vaccination status, ARDS, and patients under 18.

### Ethics declarations

The study was approved by the Institutional Research Ethics Board of the Medical Faculty of the Albert-Ludwig-University Freiburg (22–1046-retro). The requirement for informed consent was waived by the Ethics Committee of the Medical Faculty of the Albert-Ludwig-University Freiburg due to the study’s retrospective nature (22–1046-retro). The requirements of the Helsinki declaration on human research were met.

### Vaccination status

Vaccination status was divided into three categories: non-vaccinated, partially vaccinated, and fully vaccinated. Patients with explicitly recorded missing vaccination against COVID-19 and a positive COVID-19 RT-PCR test result were defined as “non-vaccinated.” Patients with a record of only one vaccine dose or diagnosed with COVID-19 infection less than 14 days after receipt of the second vaccine dose were categorized as partially vaccinated. Patients with a positive COVID-19 RT-PCR test result/onset of symptoms at least 14 days after receipt of the second vaccine dose were defined as fully vaccinated. Patients with no record of missing or receiving vaccination, and patients with missing information on the last vaccination date were categorized as having “unknown vaccination status” and excluded from the study cohort. Regarding categorization, no difference was made between the varying vaccines (ChAdOx1 nCoV-19 vaccine-AstraZeneca, BNT162b2vaccine-Pfizer-BioNTech, mRNA-1273 vaccine-Moderna), with one exception. Patients who had received the Ad26.COV2.S vaccine-Johnson& Johnson-Janssen as the first vaccine, required a second dose with an mRNA vaccine at least 14 days before a positive COVID-19 RT-PCR test result/onset of symptoms.

### Data collection-demographic and clinical parameters

After identifying the study cohort in the electronic hospital information system and the radiological information system of the study site, demographic and clinical data were extracted from electronic patient records.

Vaccination status, general information (age and sex), infection parameters (virus variant, symptom onset), symptoms (dyspnea, cough, fever), pre-existing conditions (BMI, pre-existing diseases), treatment of COVID-19 infection (pharmacological treatment, every kind of oxygen therapy such as oxygen nasal cannula, non-invasive ventilation, high flow oxygen therapy, and intubation, intensive care treatment), and complications (pulmonary superinfection, pulmonary artery embolism, exitus letalis, length of hospital stay) were recorded.

### CT examination

At the study site patients with COVID-19 infection only received a CT examination in the case of therapeutic consequences, e.g. suspicion of pulmonary superinfection or pulmonary embolism, or at admission in case of an unclear clinical constellation. All patients received a high-resolution CT using Siemens Somatom Definition Flash (Siemens Healthineers, Erlangen, Germany). Only the first acquired CT scan was evaluated in cases with follow-up CT scans. For acquisition, we used tube current modulation CARE Dose4D at quality reference mAs of 100mAs and automatic tube voltage setting with CARE kV at 120 kV reference with a collimation of 128 × 0.6 mm. Application of intravenous contrast agent depended on the clinical question that needed to be answered: native scans were performed for the extent of infection and suspicion of pulmonary superinfection, CT-pulmonary angiogram (bolus-triggered with the region of interest placed in the pulmonary trunk) was acquired while suspecting pulmonary embolism. The venous phase was acquired when superinfection with other possible focus of infection was suspected. Since the time of CT examination varied in relation to the time of infection, patients were divided into four categories, in which the CT scans were related to symptom onset: early stage (CT scan 0–5 days after symptom onset), progressive stage (CT scan 5–8 days after symptom onset), peak stage (CT scan 9–13 days after symptom onset) and late stage (CT scan ≥ 14 days after symptom onset)^30^.

### CT analysis

CT images were reviewed with the Picture Archiving and Communication System (PACS). All CT scans were analyzed in an axial reconstructed view with an image resolution of 1 mm slice thickness and in lung window (W:1500 L:-600). CT images were evaluated by two independent and blinded readers (PA; board certified radiologist with eight years of experience; EA, radiology trainee with three years of experience). PA performed image analysis of the whole study cohort, and for inter- and intrareader variability, EA performed image analysis for interreader variability). Readers were blinded to clinical data, vaccination status, virus variant, and stage of infection.

### Qualitative pattern analysis and scoring

For each CT scan, the pneumonia pattern was classified based on the RSNA Expert Consensus Statement as typical, indeterminate, atypical, and negative for pneumonia^5^. The CO-RADS and COV-RADS classification systems, which suggest the level of suspicion of COVID-19 pneumonia from very low or category 1 to very high or category 5, were additionally scored^15,16^.

The lung involvement was further qualitatively scored for the extent of lung involvement (single lobe, unilateral multilobar, and bilateral) and the axial and craniocaudal distribution of pneumonia. The predominant pneumonia pattern (GGO, consolidation, mixed or fibrotic) was analyzed. An additional note was made of the morphology of these features (rounded, subpleural, non-rounded & non-subpleural). The presence of a crazy-paving pattern, reticulation, bronchiectasis, bronchial wall thickening and nodules were documented. The presence of cavitation, lobar pneumonia, bronchoaerogram, vacuolar sign, organizing pneumonia, reverse halo sign, emphysema, and coronary calcification were noted.

### Semi-quantitative scoring

The general extent of the pneumonia was semi-quantitatively scored based on scoring systems described previously with each lobe of the lung scored separately. To summarize, the volume of involvement of each lobe was scored as 1 when less than 1/3rd of parenchyma was involved, a score of 2 was given for involvement of 1/3rd to 2/3rd of lobar volume and 3 when more than 2/3rd of the lobar volume was affected, maximum possible score for both lungs being 15^34,35^. Similarly, the total extent of GGO and consolidation was recorded.

### Inter- and intrareader variability

Inter- and intrareader variability were assessed in a random subset of 30 patients. Imaging analysis was performed with a time interval of at least three months to avoid recall bias. Inter- and intrareader variability was evaluated regarding the different scoring systems of lung changes (RSNA score, CO-RAD score, COV-RAD score), and for semi-quantitative scoring of CT-graphic pulmonary manifestations.

### Statistical analysis

Demographic and clinical parameters and qualitative and semi-quantitative data of chest CT evaluation according to vaccination status are presented as arithmetic mean and standard deviation (SD) for continuous variables and as counts and percentages for categorical variables. Overall, differences in continuous variables were evaluated by t-test and differences in categorical variables by Fisher’s Exact Test.

A multiple logistic regression with outcome CT-graphic pulmonary manifestations was calculated with step-wise adjustment to determine associations of vaccination status with CT-graphic pulmonary manifestations. Model 1 was adjusted for age and pre-existing diseases, Model 2 was additionally adjusted for virus variant, and Model 3 was additionally adjusted for the stage of infection. Relative Risks (RR) with corresponding 95% confidence intervals (CI) were calculated based on the odds ratio and the incidence of the outcome in the non-vaccinated group based on Zhang and Yu’s method^36^ (ref: Vaccinated).

When necessary, QQ-plots were used to test for normality, Levene's tests for the homogeneity of variances, correlation coefficients to check for multicollinearity, a visual check for linearity of independent variables, and log odds and cook’s distance to check for strong influential outliers. Because patients were randomly selected, independence was assumed. Failure to fulfill parametric assumptions led to the using Wilcoxon Rank-Sum and Signed-Rank tests.

Krippendorff's alpha reliability estimate was used to assess inter- and intrareader variability. Krippendorff's alpha value α ≥ 0.667 indicated acceptable reliability, and Krippendorff's alpha value α ≥ 0.800 indicated high reliability.

All analyses were conducted with R 4.2.0^37^. *P*‐values < 0.05 are considered to denote statistical significance.

## Data Availability

The data presented in this study are available on request from the corresponding author.
